# New Equivalent Electrical Model of a Fuel Cell and Comparative Study of Several Existing Models with Experimental Data from the PEMFC Nexa 1200 W

**DOI:** 10.3390/mi12091047

**Published:** 2021-08-30

**Authors:** Fatima Zahra Belhaj, Hassan El Fadil, Zakariae El Idrissi, Abdessamad Intidam, Mohamed Koundi, Fouad Giri

**Affiliations:** 1 ISA Laboratory ENSA, Ibn Tofail University, Kénitra 14000, Morocco; intidam.abdessamad@gmail.com (A.I.); mohamed.koundi@uit.ac.ma (M.K.); 2 LAC Lab, University of Caen Normandie (UNICAEN), 14000 Caen, France; fouad.giri@unicaen.fr

**Keywords:** fuel cell, new equivalent electrical model of fuel cell, PEMFC NEXA 1200, comparative study, experimental validation

## Abstract

The present work investigates different models of polymer electrolyte membrane fuel cell. More specifically, three models are studied: a nonlinear state-space model, a generic dynamic model integrated into MATLAB/Simulink, and an equivalent RC electrical circuit. A new equivalent electrical RL model is proposed, and the methodology for determining its parameters is also given. An experimental test bench, based on a 1200-W commercial PEMFC, is built to compare the static and dynamic behaviour of the existing models and the proposed RL model with the experimental data. The comparative analysis highlights the advantages and drawbacks of each of these models. The major advantages of the proposed RL model lie in both its simplicity and its ability to provide a similar transitory behaviour compared to the commercially manufactured PEMFC employed in this research.

## 1. Introduction

Today, protecting our planet is a major issue that involves several policies pertaining to transport and energy. Respecting the Kyoto Protocol and Paris Agreement on the reduction of greenhouse gas emissions, and keeping a global temperature rise this century below two degrees Celsius, requires drastic measures in favour of energy savings and the development of renewable energy. Indeed, given the increase in the global population, attention is being paid to the fact that energy supplies are necessarily limited, and that the risk of one day being faced with an energy shortage may become a reality. The transport sector today is seriously threatened because it is, on the one hand, extremely dependent on oil and, on the other hand, it is partly responsible for greenhouse gas emissions. In this respect, the use of fuel cells (FCs) in a traction system of electric vehicles is a hopeful solution, because it ultimately promises zero pollution [1]. In addition, the hydrogen sector has the advantage of being able to reduce the dependence of the transport sector on fossil fuels. Fuel cell electric vehicles (FCEVs) are classified as zero-emission vehicles (ZEVs) because they only release water. Therefore, hydrogen fuel cells have been targeted for their potential to contribute to decarbonization in the transportation sector [2,3]. The first FCEVs, which use polymer electrolyte membrane fuel cells (PEMFCs), were introduced in 2013 [4,5]. The advantages of these vehicles relative to current battery electric vehicles (BEVs) include higher driving ranges (over 500 km) and faster refuelling (3–5 min to refill the hydrogen storage tank).Therefore, the PEMFC is the essential choice for developing distributed generation power systems, hybrid electric vehicles, and other emerging fuel cell applications. It is therefore important for electrical and automation engineers and researchers to understand the dynamic behaviour of the PEM fuel cell for its successful use in different applications. In the literature, many research works are attempts to develop models of the PEM fuel cell.

In the beginning, electrochemistry-based models of the PEM fuel cell were introduced [6,7]. Then, dynamic models started to emerge [8,9,10,11,12]. In [13] a dynamic model of PEMFC, using the exact linearization approach, was presented. Although these models provide a certain understanding of the PEMFC, they remain insufficient to design adequate controllers for PEM fuel cell systems. It is for this reason that state-space models were introduced in some works [14,15]. However, state-space models are further complicated because they are highly nonlinear, and involve a large number of state variables and parameters. Then, some works have attempted to develop equivalent electric models, because they are still simple and easy to understand and to implement [16,17,18,19,20,21,22,23,24,25].

The present work investigates different classes of models proposed in the literature. More specifically, three models are presented: a nonlinear state-space model, a generic dynamic model integrated into MATLAB/Simulink, and an equivalent electric RC circuit. Using the dynamic behaviour of a 1200-W commercialized PEMFC, a new equivalent electric model is proposed. A comparative study between the proposed model and the previous models is conducted, showing the pros and cons of each model.

The rest of the paper is organized as follows: In Section 2, the electrochemical principle of a PEMFC is presented. Section 2.1 is devoted to the presentation of the nonlinear state-space model. A generic dynamical model integrated into MATLAB/Simulink is illustrated in Section 2.2. In Section 2.3, an equivalent electrical RC circuit is presented. The proposed equivalent electrical RL circuit is shown in Section 2.4. Section 3. is devoted to the experimental behaviour of a 1200-W commercialized PEMFC. The comparative study between different models is conducted in Section 4. A conclusion and a reference list end the paper.

## 2. Theoretical Principle

**Figure 1:**
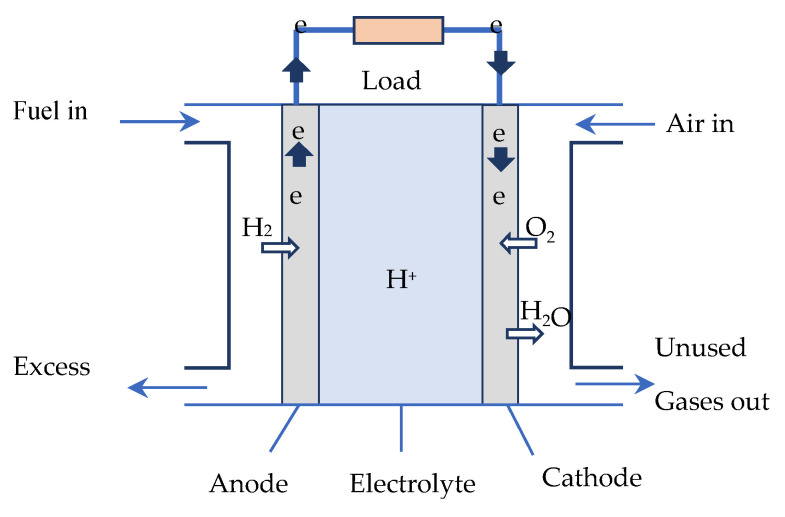
Schematic diagram of a PEMFC.

A fuel cell (FC) is an electrochemical energy generator used to directly transform the chemical energy of a fuel (hydrogen, hydrocarbons, alcohols, etc.) into electrical energy. Figure 1 shows a schematic of a hydrogen PEMFC. The FC core consists of three elements, including two electrodes—an oxidizing anode (electron emitter), and a reducing cathode (electron collector)—separated by an electrolyte. The FC is supplied by an injection of hydrogen at the anode and air at the cathode. Continuous electrical energy is then available across the FC.

In the core of a hydrogen fuel cell of the PEMFC type, two electrochemical reactions occur successively [26,27]:

At the anode: catalytic oxidation of the hydrogen, which dissociates from its electrons:

(1) $$H_{2}\to 2H^{+}+2e^{\_}$$

At the cathode: catalytic reduction of the oxygen, which captures the *H*^+^ ions that have passed through the electrolyte membrane, as well as the electrons arriving from the external circuit. The reaction produces heat and water:

(2) $$\frac{1}{2}O_{2}+2H^{+}+2e^{\_}\to H_{2}O+Q\left(heat\right)$$

**Figure 2:**
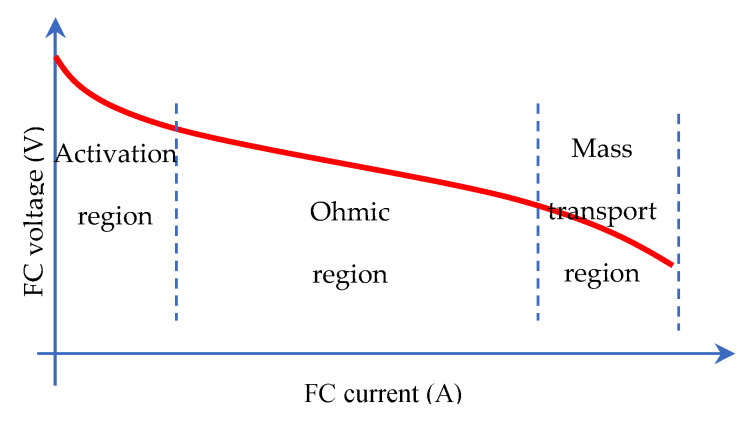
Nonlinear *i*–*v* characteristic of the fuel cell.

To evaluate the PEMFC’s performance, and for control purposes, several mathematical models of PEMFC have been developed in the literature. They can be classified into three main categories:

Static models representing the input–output behaviour of the FC—in particular, the nonlinear current–voltage characteristic (see Figure 2). The output voltage of the fuel cell is dependent on the thermodynamically predicted fuel cell voltage output, and three major losses: activation losses (due to the electrochemical reaction), ohmic losses (due to the ionic electronic condition), and concentration losses (due to mass transport).

Nonlinear state-space representing the internal behaviour of the fuel cell and equivalent electrical circuits.

In this paper a comparison between four models is investigated: a nonlinear state-space model, a generic model from MATLAB Toolbox, an equivalent electrical circuit RC, and a new proposed equivalent electrical circuit RL. The resulting models will be compared to the experimental results using a 1.2-kW fuel cell module from Ballard (the Nexa 1200).

### 2.1. Nonlinear State-Space Model of the PEMFC (NLM)

It has been suggested in many studies [12,15,16,28] that a nonlinear state-space model of the PEM fuel cell could be represented by Equations (3)–(8). In this model, the open-circuit output voltage of the PEM fuel cell, mass balance and thermodynamic energy balance, irreversible voltage losses, and the formation of the charged double layer in the PEM fuel cell is modelled:

(3) $${\dot{x}}_{1}=-\theta_{1}x_{1}+\theta_{1}u_{T_{R}}-L\left(x\right)I_{fc}$$

(4) $${\dot{x}}_{2}=2\theta_{2}x_{1}u_{P_{A}}-2\theta_{2}x_{1}x_{2}-\theta_{3}x_{1}I_{fc}$$

(5) $${\dot{x}}_{3}=2\theta_{4}x_{1}u_{P_{C}}-2\theta_{4}x_{1}x_{3}-\theta_{5}x_{1}I_{fc}$$

(6) $${\dot{x}}_{4}=2\theta_{6}x_{4}x_{1}+2\theta_{5}x_{1}I_{fc}$$

(7) $${\dot{x}}_{5}=-\theta_{5}x_{5}+\theta_{6}I_{fc}$$

where $x_{1}=T$ is a stack temperature; $x_{2}=P_{H_{2}}$ is the partial pressure of hydrogen; $x_{3}=P_{O_{2}}$ is the partial pressure of oxygen; $\;x_{4}=P_{H_{2}O}$ is the partial pressure of water; $x_{5}=V_{fc}$ is the output voltage of the PEM fuel cell; $I_{fc}$ is the stack current; $u_{P_{A}}$ is the channel pressure of hydrogen; $u_{P_{C}}$ is the channel pressure of oxygen; $u_{T_{R\;}}$ is room temperature; and the involved parameters and functions are given as follows:

$$L\left(x\right)=n_{s}\left[\left(\frac{2E_{0}^{Cell}}{M_{fc}C_{fc}}\right)+\left(\frac{Rx_{1}}{FM_{fc}C_{fc}}\right)\ln\left(\frac{x_{2}x_{3}^{0.5}}{x_{4}}\right)-V^{Act}-V^{Conc}-V^{O}\right]$$

$$\theta_{1}=\frac{h_{s}n_{s}A_{s}}{M_{fc}C_{fc}}$$

θ2=[(R(mH2O)inax1)(Va(PH2O)ina)]

$$\theta_{3}=\left[\frac{Rx_{1}}{4V_{c}F}\right]$$

$$\theta_{4}=\left[\frac{\left(R{\left(m_{H_{2}O}\right)}_{in}^{c}x_{1}\right)}{\left(V_{c}{\left(P_{H_{2}O}\right)}_{in}^{c}\right)}\right]$$

$$\theta_{5}=\left[\frac{Rx_{1}}{4V_{c}F}\right]$$

$$\theta_{6}=\left[\frac{\left(R{\left(m_{H_{2}O}\right)}_{in}^{c}\left(P_{H_{2}O}^{in}-x_{4}\right)\right)}{\left(V_{c}{\left(P_{H_{2}O}\right)}_{in}^{c}\right)}\right]$$

$$\theta_{7}=\frac{1}{C\left(R_{ac}+R_{co}\right)}$$

(8) $$\theta_{8}=\frac{1}{C}$$

### 2.2. Generic Model from MATLAB Toolbox (GMM)

**Figure 3:**
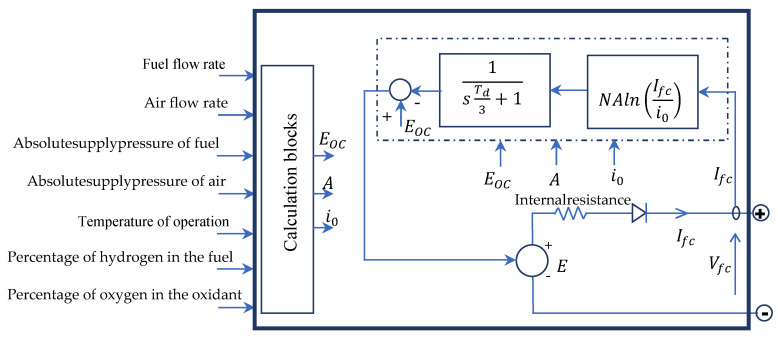
Circuit of the generic model of a fuel cell stack.

A fuel cell stack block integrated into MATLAB/Simulink implements a generic model parameterized to represent the most popular types of fuel cell stacks fed with hydrogen and air. The block represents two versions of the stack model: a simplified model, and a detailed model. The user can switch between the two models by selecting the level in the mask under the model detail level in the block dialogue box. In this paper, we consider the detailed model represented by Figure 3. The notations used are the same as those from [29,30].

The fuel cell voltage is related to the fuel cell current as follows:

(9) $$V_{fc}=E-R_{i}\times I_{fc}$$

where $R_{i}$ is the internal resistance, and the controlled voltage source *E* is described by the following equation:

(10) $$E=E_{oc}-NAln\left(\frac{I_{fc}}{i_{0}}\right)\times \frac{1}{s\frac{T_{d}}{3}+1}$$

where *s* is the Laplace operator and $E_{oc}$ is an open circuit voltage (V); *N* is the number of cells; *A* is a Tafel slope (V); *i_0_* is the exchange current (A); and *T_d_* is the response time (at 95% of the final value) (s). In Equation (10), the parameters ($E_{oc}$, N, $i_{0}$) are updated online based on the input pressures and flow rates, stack temperature, and gas compositions [29].

### 2.3. Equivalent Electrical RC Circuit (RCM)

**Figure 4:**
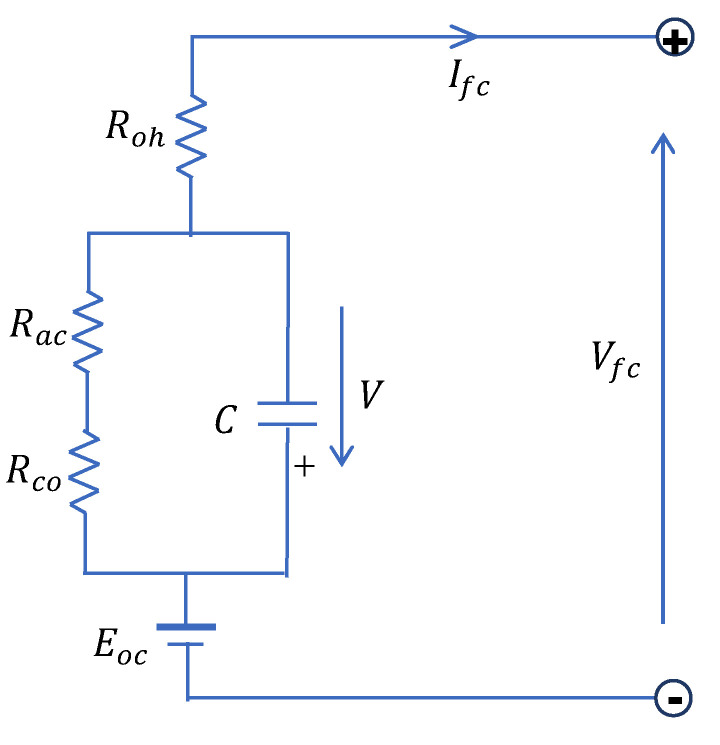
Equivalent electric RC circuit of a fuel cell stack.

Most dynamic models for PEMFCs are complex, and are not easy to use for control purposes. An equivalent electrical circuit could be used as a good alternative to model the fuel cell’s dynamical behaviour as represented in Figure 4.

From this figure, the fuel cell stack’s static electrochemical behaviour can be represented by the following equations [15]:

(11) $$V_{fc}=E_{oc}-V-R_{oh}I_{fc}$$

(12) $$\frac{dV}{dt}=\frac{1}{C}I_{fc}-\frac{1}{\tau }V$$

where *V* represents the dynamical voltage across the equivalent capacitor; *C* is the equivalent electrical capacitance; $R_{oh}$ is the ohmic resistance; and τ  is the fuel cell electrical time constant, defined as follows:

(13) $$\tau =\left(R_{ac}+R_{co}\right)C$$

In Equation (11), $E_{oc}$ is the open-circuit voltage, defined as follows:

(14) $$E_{oc}=n_{s}\left(E_{Nernst}-V_{act}\right)$$

where $\;n_{s}$ is the number of cells in series in the stack; $E_{Nernst}$ is the thermodynamic potential of the cell, and represents its reversible voltage or Nernst potential; and $V_{act}$ is the activation voltage drop. The quantities $E_{Nernst}V_{act}$ are given as follows [15,16,21,30]:

(15) $$\begin{array}{cc} E_{Nernst}= & 1.229-8.5\times 10^{-4}\times \left(T-298.15\right)-3.33\times 10^{-3}I_{fc}\left(s\right)\frac{80s}{80s+1}\\ & +4.31\times 10^{-5}\times T\times \left(\ln\left(P_{H2}\right)+\frac{1}{2}\left(P_{O2}\right)\right)-3.33\times 10^{-3}I_{fc}\left(s\right)\frac{80s}{80s+1} \end{array}$$

(16) $$V_{act}=-0.948+T\times \left[2.86\times 10^{-3}+2\times 10^{-4}\ln\left(A\right)+4.3\times 10^{-5}\ln\left(C_{H_{2}}\right)+7.6\times 10^{-5}\ln\left(C_{O_{2}}\right)\right]$$

where $P_{H_{2}}$ and $P_{O_{2}}$ are the partial pressures (atm) of hydrogen and oxygen, respectively; *T* is the cell’s absolute Kelvin temperature; and *A* is the cell’s active area (cm^2^). The terms$\;C_{O_{2}}$ and $C_{H_{2}}$ presented in Equation (16) are the oxygen concentration at the cathode membrane/gas interface (mol/cm^3^), and the liquid phase concentration of hydrogen at the anode/gas interface (mol/cm^3^), respectively. They can be obtained as follows [21]:

(17) $$C_{O_{2}}=\frac{P_{O_{2}}}{5.08\times 10^{6}\exp\left(\frac{-498}{T}\right)}$$

(18) $$C_{H_{2}}=\frac{P_{H_{2}}}{1.09\times 10^{6}\exp\left(\frac{77}{T}\right)}$$

**Figure 5:**
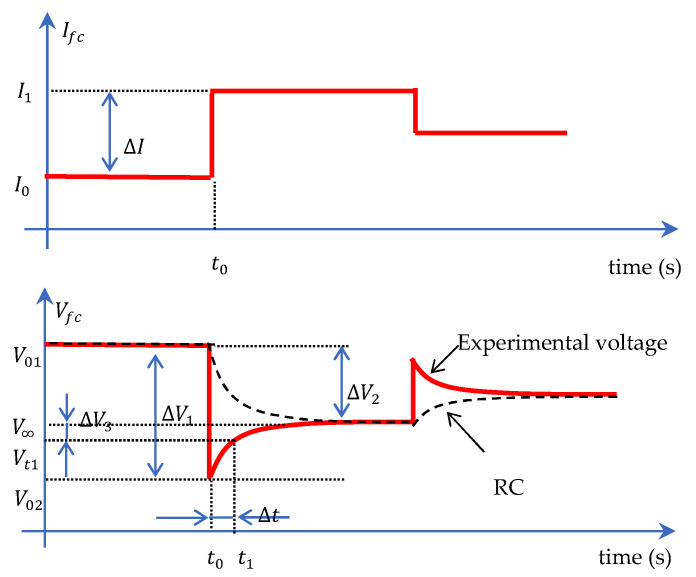
Comparison between the shape of the experimental FC voltage with the equivalent RC circuit.

It should be emphasized that the capacitor *C* in the RC model of Figure 4 affects the transient response of the PEMFC. Using the simulation RC model shown in Figure 4, the shape of the transient response of this model to the step load change is represented in Figure 5. It should be noted that when the load current steps up, the voltage drops simultaneously to some value due to the ohmic losses, and then it decays exponentially to its steady-state value due to the capacitor *C*. However, the experimental voltage of the PEMFC, as can be seen later (Section 5) and found in many works [12,31,32] has the form of Figure 5. This figure clearly illustrates a big difference between the experimental fuel cell voltage and the corresponding voltage given by the RC model. We conclude that the equivalent electrical RC circuit is not suitable for a PEM fuel cell. In the next section, we will present a new equivalent electrical model using an inductor instead of a capacitor, and we will show that the transient of the obtained model fits the experimental transient.

### 2.4. Equivalent Electrical RL Circuit (RLM)

#### 2.4.1. Proposed RL Circuit

**Figure 6:**
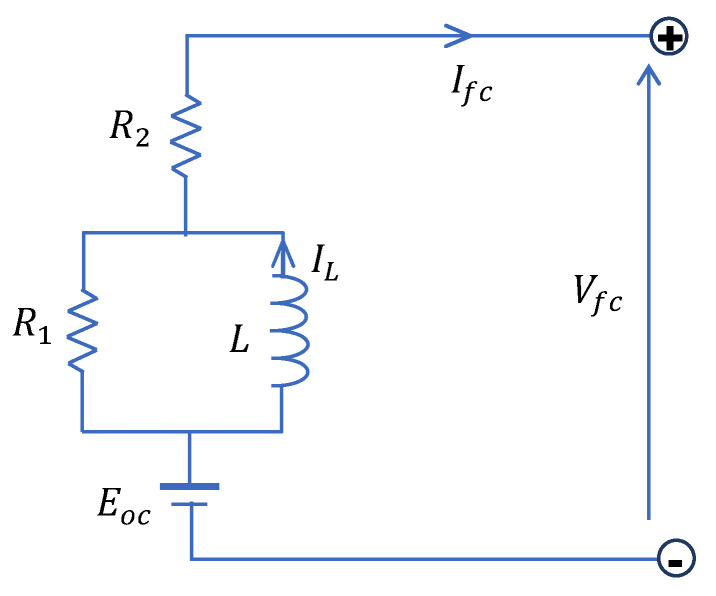
Equivalent electric RL circuit of a fuel cell stack.

As shown in the previous section, the equivalent electrical RC model is not appropriate for modelling the dynamics of a fuel cell, since its transient is different compared to the experimental data. In this paper, a new equivalent electrical circuit is proposed using an inductor and resistors, as shown in Figure 6. Note that the open-circuit voltage $E_{oc}$ remains the same as in Equation (14).

#### 2.4.2. Determination of Model Parameters

The electrical model presented in Figure 6 involves three parameters$—L,\;\;R_{1},$ and $R_{2}$—which can be determined using the response of the fuel cell voltage to load current step change. Let us first introduce some useful electrical relationships based on the proposed circuit.

The fuel cell voltage is governed by the following equations:

(19) $$V_{fc}=E_{oc}-R_{2}I_{fc}-R_{1}\left(I_{fc}-I_{L}\right)$$

(20) $$L\frac{dI_{L}}{dt}=R_{1}\left(I_{fc}-I_{L}\right)$$

(21) $$L\frac{dI_{L}}{dt}+R_{1}I_{L}=R_{1}I_{fc}$$

For $t<t_{0}$ we suppose that $\;I_{fc}=I_{0}=cte,$ which corresponds to a constant fuel cell voltage $V_{fc}=V_{0}=cte$ (see Figure 5). At the instant $t=t_{0}$, we apply a current step change from $I_{0}$ to $\;I_{1}$, and then the inductor current $I_{L}$ will evolve, using Equation (21), according to the following equation:

(22) $$I_{L}=I_{1}+\left(I_{0}-I_{1}\right)e^{-\frac{\left(t-t_{0}\right)}{\tau_{L}}}$$

where $\;\tau_{L}=\frac{L}{R_{1}}$ is a time constant of the RL circuit. Moreover, using Equations (19) and (22), the voltages $\;V_{01}$, $V_{02},\;$and $V_{\infty \;}$ represented in Figure 5 are given as follows:

$$V_{01}=E_{oc}-R_{2}I_{0}$$

(23) $$V_{02}=E_{oc}-R_{2}I_{1}-R_{1}\left(I_{1}-I_{0}\right)$$

$$V_{\infty }=E_{oc}-R_{2}I_{1}$$

It follows that the voltage variations $\Delta V_{1}$ and $\Delta V_{2}$, corresponding to the current variation $\Delta I=I_{1}-I_{0}$, are given by:

(24) $$\Delta V_{1}=V_{01}-V_{02}=\left(R_{1}+R_{2}\right)\left(I_{1}-I_{0}\right)=\left(R_{1}+R_{2}\right)\Delta I$$

(25) $$\Delta V_{2}=V_{01}-V_{\infty }=R_{2}\left(I_{1}-I_{0}\right)=R_{2}\Delta I$$

Now, taking any instant $\;t_{1}>t_{0}$ in the transient of the fuel cell voltage, using Equations (19), (22), and (23), one has:

(26) $$V_{t1}=V_{\infty }-R_{1}\left(I_{1}-I_{0}\right)e^{-\frac{\left(t_{1}-t_{0}\right)}{\tau_{L}}}$$

which, in turn, taking into account Equations (24) and (25), gives:

(27) $$\Delta V_{3}=V_{\infty }-V_{t1}=R_{1}\Delta Ie^{-\frac{\Delta t}{\tau_{L}}}=\left(\Delta V_{1}-\Delta V_{2}\right)e^{-\frac{\Delta t}{\tau_{L}}}$$

where $\;\Delta t=t_{1}-t_{0}$.

Finally, the procedure for determining the RL model parameters can be summarized as follows:

1. From the plot of the fuel cell voltage corresponding to any current step change from $I_{0}$ to $I_{1}$, determine  ΔI, $V_{01}$, $V_{02},$ and $V_{\infty }$;
2. Take any instant $t_{1}>t_{0}$ in the transient and determine its corresponding voltage $V_{t1}$ and $\Delta t=t_{1}-t_{0}$;
3. Calculate, using Equations (24), (25), and (27): $\;\Delta V_{1}$, $\Delta V_{2}$, and $\Delta V_{3}$, respectively;
4. Calculate $R_{2}$ using Equation (25): $R_{2}=\frac{\Delta V_{2}}{\Delta I}$;
5. Calculate $R_{1}$ using Equation (24): $R_{1}=\frac{\Delta V_{1}}{\Delta I}-R_{2}$;
6. Calculate the inductance value *L*, using Equation (27), as follows: $L=\frac{\Delta t\times R_{1}}{\ln\left(\frac{\Delta V_{1}-\Delta V_{2}}{\Delta V_{3}}\right)}$

## 3. Experimental Model (EXM)

**Figure 7:**
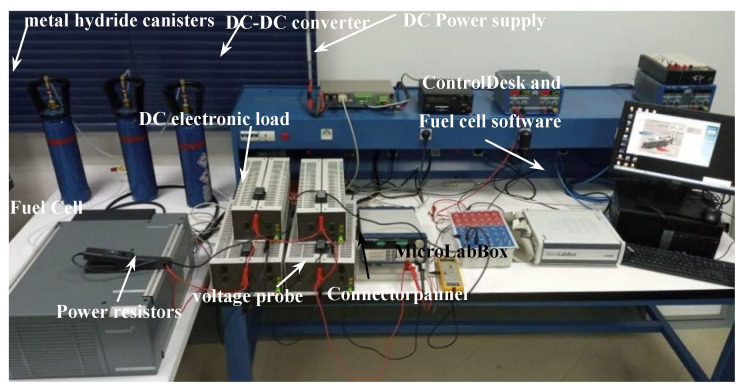
View of the experimental bench.

In this section, we will determine the static and the dynamic behaviour of the Ballard Nexa 1200 fuel cell module, which has a rated power of 1.2 kW. To this end, an experimental bench was built, as shown in Figure 7; it consists—in addition to the fuel cell, with its monitoring software—of three metal hydride canisters from Heliocentris with storage capacities of 800 NL hydrogen, an H2 connection Kit 15 bar for connecting the metal canisters, a Nexa 1200 DC/DC converter, a power supply from BK Precision used for the fuel cell starter, Hall effect sensors to measure the voltage and current variables, a programmable DC electronic load from BK Precision and power resistors to make load changes, and a MicroLabBox-dSPACE with Control Desk software plugged into a personal computer for signal acquisition.

### 3.1. Static Characteristics(i–v) of the Fuel Cell

**Figure 8:**
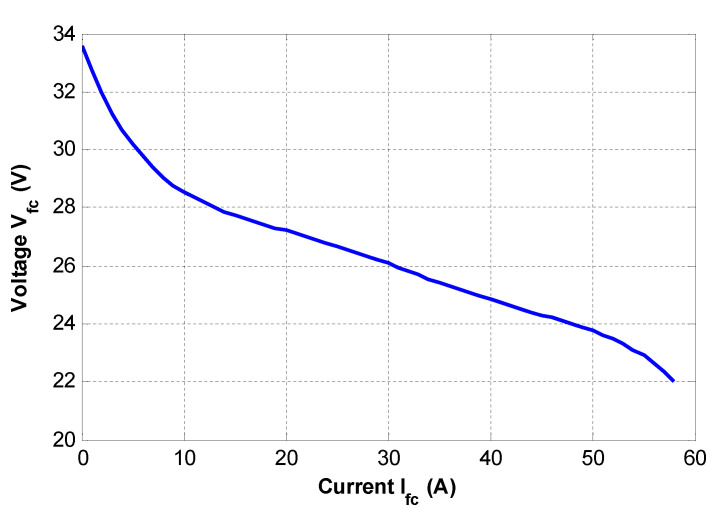
Obtained experimental (*i–v*) characteristics of the Nexa 1200 fuel cell module.

Several points representing the current and the voltage under static conditions of the fuel cell were determined, and are listed in Table 1. The obtained current–voltage characteristics are illustrated by Figure 8.

### 3.2. Dynamic Behaviour of the NEXA 1200Fuel Cell

**Figure 9:**
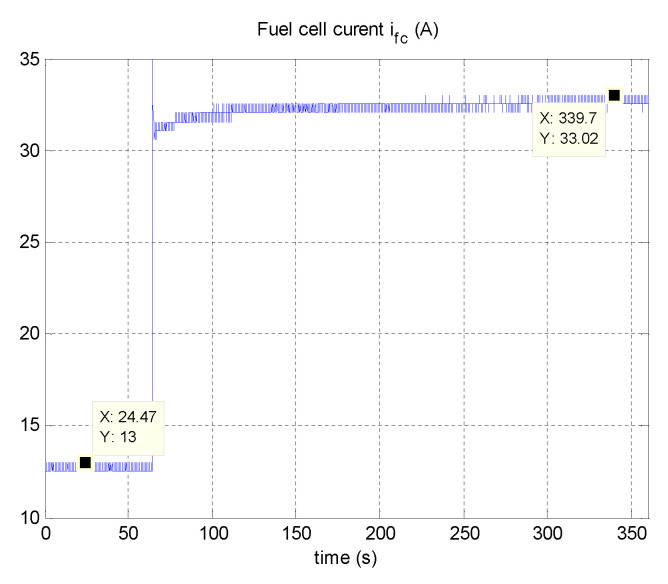
Fuel cell current step change.

Using a programmable DC electronic load and power resistors, a fuel cell current step change is operated from 13A to 33A after 65.1 s. The resulting fuel cell current and voltage are shown in Figure 8 and Figure 9, respectively.

### 3.3. RL Model Parameters

**Figure 10:**
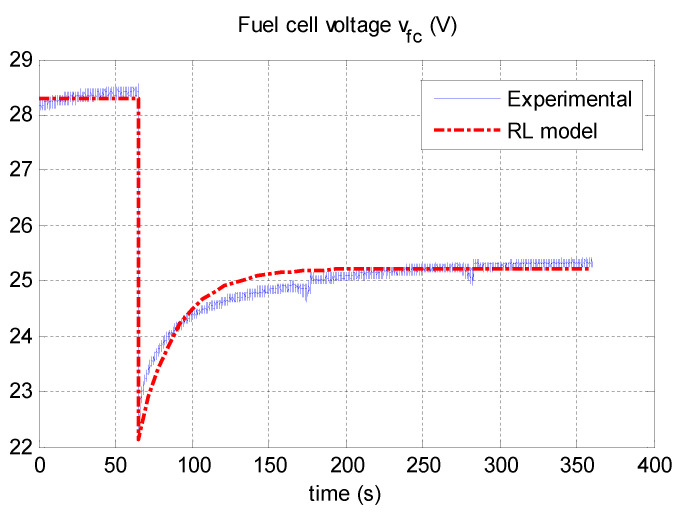
Comparison between the experimental and RL models.

Using the procedure shown in Section 2.4, which describes the method for determining the parameters of the equivalent electric model RL, and using the experimental responses shown in Figure 8 and Figure 9, we obtained the results listed in Table 2. Figure 10 shows a comparison between the experimental voltage of the fuel cell and that obtained from the RL model. One can show a good fit for the proposed RL model.

## 4. Comparison between Different Models

**Figure 11:**
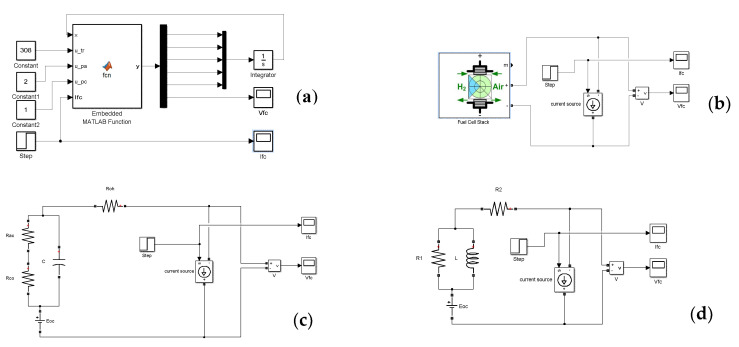
Simulated models in MATLAB/Simulink: (**a**) nonlinear model (NLM); (**b**) generic MATLAB model (GMM); (**c**) RC model (RCM); and (**d**) RL model (RLM).

In this section, we will evaluate the static and the dynamic behaviour of the studied models, and compare them to the experimental data of the used Nexa 1200 fuel cell module. All models are simulated using MATLAB/Simulink software. As a load of these models, we used a controlled current source whose variations were programmed similarly to those used for the experiment. Figure 11 illustrates the simulated models, while the experiments were carried out according to Figure 7. All of the parameters used for the simulation models are listed in Table 3.

### 4.1. Static Behaviour

**Figure 12:**
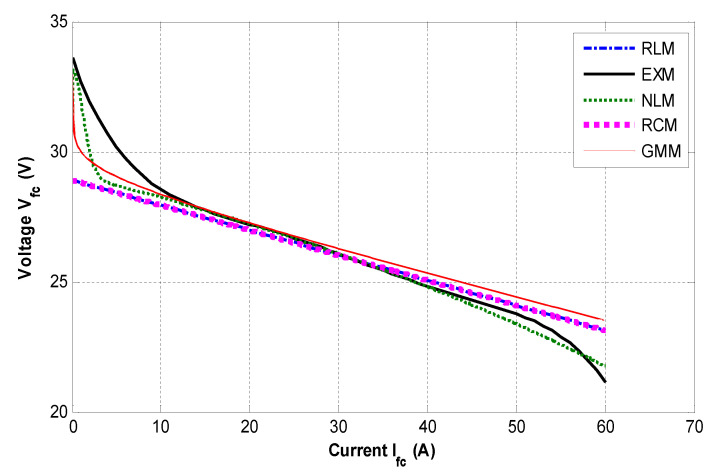
*i–v* characteristics of simulated models compared to the experiments.

The static behaviour of the simulated models is compared to the experimental results. Figure 12 illustrates the obtained current–voltage characteristics.

To compare between different models, the following root-mean-square error (RMSE) criterion is selected, which is a frequently used measure of the differences between values predicted by a model and the values observed:

(28) $$RMSE=\sqrt{\frac{\sum_{k=1}^{N}{\left(V_{fcx}\left(k\right)-V_{fcm}\left(k\right)\right)}^{2}}{N}}$$

where $(V_{fcx}-V_{fcm})\;$ is the error between the measured (experimental) and the modelled fuel cell voltage; and *N* is the total number of samples. The obtained results are summarized in Table 4. It is evident from the table that the nonlinear model is a better model; however, it requires a longer computational time, which is considered a drawback for real-time application and control purposes. The classic RC model and the proposed RL model have practically the same RMSE in static conditions. Nevertheless, we will later see the great supremacy of the proposed RL model in a dynamic regime.

### 4.2. Dynamic Behaviour

**Figure 13:**
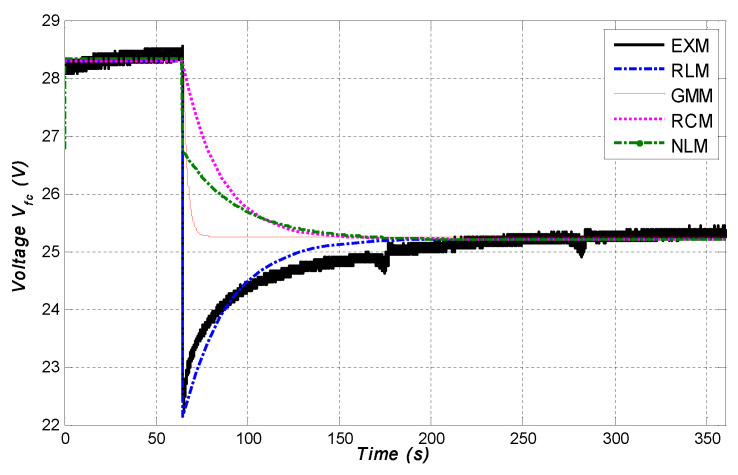
Dynamic behaviour of simulated models compared to the experiments.

In this section, a comparison between the dynamic behaviour of four models and the experimental results in the presence of fuel cell current changes was studied. Figure 13 illustrates the dynamic behaviour of each model. As is clearly shown, the proposed RL equivalent electrical model presents the best dynamic behaviour compared to the conventional RC model used in the literature. The main advantages of this model lie in its simplicity and stability to produce the same behaviour as the fuel cell.

Unlike the proposed RL equivalent electrical model, the mathematical model, the conventional generic MATLAB model and the RL model all provide the same static behaviour of the fuel cell, but do not produce a smooth transitory fuel cell behaviour. In addition, the disadvantages of the mathematical model are that it is complicated and requires a longer computational time for calculation. Consequently, this is a handicap for real-time control and implementation.

Therefore, the present modelling approach permits the researchers and students who do not own a real fuel cell to approximate its static and dynamic behaviour in a simple, fast, and affordable way.

## 5. Conclusions

The fuel cell is an electric component that is used more and more in distributed generation power fields, but also in hybrid electric vehicles. Its modelling is an important step not only to understand its dynamic behaviour, but also to develop advanced controllers for systems based on the fuel cell. Several models are proposed in the literature; this work re-investigates some of these most common models. The study shows that the equivalent electric RC circuit is not appropriate, since it presents a different behaviour from the experimental results. Therefore, the paper presents an analysis of a new equivalent electric RL circuit. A comparative study of different models with experimental data from a Nexa 1200 PEMFC reveals the advantages and disadvantages of each model. It turned out that the mathematical nonlinear model is better than the other investigated models, but requires a longer computational time, which is considered a drawback for real-time application and control purposes. The classic RC model and the proposed RL model have practically the same RMSE in static conditions. Nevertheless, the proposed equivalent electric RL model presents the advantages of providing the best transient behaviour compared to the classic RC model; indeed, contrary to the investigated models, the advantage of the proposed equivalent RL model lies essentially in its simplicity, and its ability to produce a transient behaviour similar to that of the commercial fuel cell used in this work.

## Figures and Tables

**Table 1:** Experimental data of the fuel cell current and voltage.

I_fc_current (A)	0	1	2	3	4	5	6	7	8	9
V_fc_voltage	33.58	32.7	31.93	31.26	30.68	30.18	29.75	29.38	29.06	28.78
I_fc_current (A)	11	12	13	14	15	16	17	18	19	20
V_fc_voltage	28.34	28.16	28	27.86	27.74	27.62	27.52	27.41	27.31	27.21
I_fc_current (A)	22	23	24	25	26	27	28	29	30	31
V_fc_voltage	27	26.9	26.79	26.68	26.56	26.44	26.32	26.19	26.07	25.94
I_fc_current (A)	33	34	35	36	37	38	39	40	41	42
V_fc_voltage	25.68	25.55	25.42	25.3	25.17	25.05	24.93	24.82	24.71	24.6
I_fc_current (A)	44	45	46	47	48	49	50	51	52	53
V_fc_voltage	24.39	24.29	24.19	24.09	23.98	23.88	23.75	23.62	23.47	23.31
I_fc_current (A)	55	56	57	58	59	60				
V_fc_voltage	22.89	22.64	22.34	22	21.6	21.13				

**Table 2:** RL circuit parameters.

ΔI (A)	$\Delta V_{1}(V)$	$\Delta V_{2}(V)$	$\Delta V_{3}(V)$	Δt (s)	$R_{1}$ (mΩ)	$R_{2\;}$ (mΩ)	L (H)
20	6.11	3.03	0.98	36.7	154	151.5	4.94

**Table 3:** Simulation parameters.

Type of Model	Parameters
Nonlinear model (NLM)	Parameters used in [1]
Generic MATLAB mode (GMM)	Voltage at 0 A and 1 A [30] Nominal operating point (52 A, 24.23 V) Maximum operating point (100 A, 20 V)
RC model (RCM)	$E_{oc}=28.32\;V$; $R_{oh}=2.89958\;m\Omega$;$\;R_{ac}+R_{CO}=155\;m\Omega$; C=130 F
Proposed RL model (RLM)	$E_{oc}=28.32\;V$; $R_{1}=157.70\;m\Omega$;$\;R_{2}=156.17\;m\Omega$; L=3.1078 H

**Table 4:** RMSE criteria for static (*i–v*) characteristic.

Type of Model	RMSE
Nonlinear model (NLM)	0.1852
Generic MATLAB model (GMM)	0.1961
RC model (RCM)	0.2382
Proposed RL model (RLM)	0.2319

## Nomenclature

$E_{0}^{Cell}$	Reference potential at standard operating conditions (V).
$h_{s}$	Convective heat transfer coefficient (W/(m^2^K)).
R	Universal gas constant (J/ (molK)).
T	Stack temperature (K).
F	Faraday’s constant (C/mol).
$P_{H_{2}}$	The partial pressure of hydrogen (atm).
$P_{O_{2}}$	The partial pressure of oxygen (atm).
$P_{H_{2}0}$	The partial pressure of water (atm).
${\left(P_{H_{2}O}\right)}_{in}^{a}$	The partial pressure of waterat the anode (atm).
${\left(P_{H_{2}0}\right)}_{in}^{c}$	The partial pressure of water at the cathode (atm).
${\left(m_{H_{2}O}\right)}_{in}^{a}$	A mole flow rate of water at the anode (mol/s).
${\left(m_{H_{2}O}\right)}_{in}^{c}$	A mole flow rate of water at the cathode (mol/s).
$V^{Act}$	Activation voltage loss (V).
$V^{Conc}$	Concentration voltage loss (V).
$V^{O}$	Ohmic voltage loss (V).
$V_{a}$	The volume of the anode (m^3^).
C	Capacitance due to the charge double layer(F): equivalent electrical capacitance
$R_{ac}$	Equivalent resistance corresponding to activation voltage loss (Ω).
$R_{co}$	Equivalent resistance corresponding to concentration voltage loss (Ω).
$R_{oh}$	Ohmic resistance (Ω).
$A_{s}$	Area of a single cell (m^2^).
$M_{fc}$	The total mass of the PEM fuel cell stack (kg).
$C_{fc}$	Specific heat capacity of the PEM fuel cell stack [J/(molK)].
$I_{fc}$	Stack current (A).
$n_{s}$	The number of PEM fuel cell stacks.
$u_{T_{R}}$	Room temperature (K).
$u_{P_{A}}$	Channel pressure of hydrogen (atm).
$V_{C}$	The voltage across the capacitor C (V).
$V_{fc}$	The output voltage of the PEM fuel cell (V)
NLM	State-space model (nonlinear model)
GMM	Generic MATLABmodel
RLM	RL model
RCM	RC model
EXM	Experimental model
